# Tri-μ-chlorido-bis­[(η^6^-hexa­methyl­benzene)­ruthenium(II)] tetra­chlorido­ferrate(III)

**DOI:** 10.1107/S160053681103621X

**Published:** 2011-09-14

**Authors:** Petr Štěpnička, Jiří Schulz, Ivana Císařová

**Affiliations:** a Department of Inorganic Chemistry, Faculty of Science, Charles University in Prague, Hlavova 2030, 12840 Prague 2, Czech Republic

## Abstract

The mol­ecular geometry of the complex cation in the title structure, [(μ-Cl)_3_{Ru^II^(η^6^-C_6_Me_6_)}_2_][Fe^III^Cl_4_], compares very well with that reported earlier for the corresponding PF_6_^−^ salt [Pandey *et al.* (1999 ▶). *J. Organomet. Chem.* **592**, 278–282]. The [FeCl_4_]^−^ counter ion has a rather regular tetra­hedral geometry with Fe—Cl distances and Cl—Fe—Cl angles in the range 2.1891 (7)–2.2018 (8) Å and 107.10 (3)–110.56 (3)°, respectively. There are no significant inter­molecular inter­actions in the crystal except for some weak C—H⋯Cl contacts, which in turn indicates that the crystal packing is determined predominantly by electrostatic inter­actions between the ionic constituents.

## Related literature

Crystals of the title compound were isolated during attempted recrystallization of [(η^6^-C_6_Me_6_)RuCl_2_{Ph_2_PfcCON(CH_2_CH_2_OH)_2_}] [fc = ferrocene-1,1′-diyl; for the preparation of this ligand, see Schulz *et al.* (2009 ▶)] from chloro­form–diethyl ether. It is likely a decomposition product as the result of photolytic cleavage of the ferrocene moiety in the halogenated solvent (Brand & Snedden, 1957 ▶). For the crystal structure of [(μ-Cl)_3_{Ru(η^6^-C_6_Me_6_)}_2_][PF_6_], see: Pandey *et al.* (1999 ▶); Redwine *et al.* (2000 ▶). For the first structurally characterized compound of this type, [(μ-Cl)_3_{Ru(η^6^-C_6_Me_6_)}_2_][BPh_4_]·CH_3_OH, see: Tocher & Walkinshaw (1982 ▶). For the structures of simple tetra­chloridoferrate(III) salts, see: Wyrzykowski *et al.* (2006 ▶); Jin *et al.* (2005 ▶). 
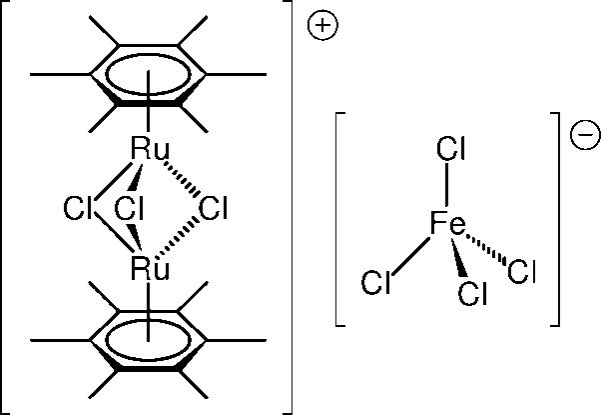


## Experimental

### 

#### Crystal data

[Ru_2_Cl_3_(C_12_H_18_)_2_][FeCl_4_]
*M* *_r_* = 830.67
Triclinic, 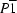

*a* = 8.4490 (2) Å
*b* = 12.8352 (2) Å
*c* = 14.6752 (4) Å
α = 106.5767 (12)°
β = 90.4341 (9)°
γ = 99.7915 (12)°
*V* = 1500.43 (6) Å^3^
*Z* = 2
Mo *K*α radiation
μ = 2.11 mm^−1^
*T* = 150 K
0.30 × 0.20 × 0.08 mm

#### Data collection

Nonius KappaCCD diffractometer
Absorption correction: Gaussian using the diffractometer software *T*_min_ = 0.529, *T*_max_ = 0.855
27082 measured reflections
6900 independent reflections
6172 reflections with *I* > 2σ(*I*)
*R*_int_ = 0.036

#### Refinement

*R*[*F* ^2^ > 2σ(*F* ^2^)] = 0.026
*wR*(*F* ^2^) = 0.061
*S* = 1.08
6900 reflections
319 parameters
H-atom parameters constrained
Δρ_max_ = 0.47 e Å^−3^
Δρ_min_ = −0.68 e Å^−3^

### 

Data collection: *COLLECT* (Nonius, 2000 ▶); cell refinement: *HKL* *SCALEPACK* (Otwinowski & Minor, 1997 ▶); data reduction: *HKL* (Otwinowski & Minor, 1997 ▶) *DENZO* and *SCALEPACK*; program(s) used to solve structure: *SIR97* (Altomare *et al.*, 1999 ▶); program(s) used to refine structure: *SHELXL97* (Sheldrick, 2008 ▶); molecular graphics: *PLATON* (Spek, 2009 ▶); software used to prepare material for publication: *SHELXL97* and *PLATON*.

## Supplementary Material

Crystal structure: contains datablock(s) I, global. DOI: 10.1107/S160053681103621X/su2311sup1.cif

Structure factors: contains datablock(s) I. DOI: 10.1107/S160053681103621X/su2311Isup2.hkl

Additional supplementary materials:  crystallographic information; 3D view; checkCIF report

## Figures and Tables

**Table 1:** Hydrogen-bond geometry (Å, °)

*D*—H⋯*A*	*D*—H	H⋯*A*	*D*⋯*A*	*D*—H⋯*A*
C9—H9*A*⋯Cl3^i^	0.96	2.80	3.629 (3)	145
C11—H11*B*⋯Cl6^ii^	0.96	2.71	3.588 (3)	153

Symmetry codes: (i) 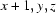; (ii) 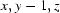.

**Fig. 1.**
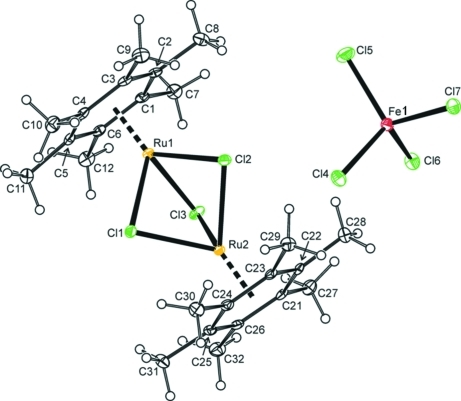
View of the molecular structure of the title compound, showing the atom numbering scheme and displacement ellipsoids drawn at the 30% probability level.

**Fig. 2.**
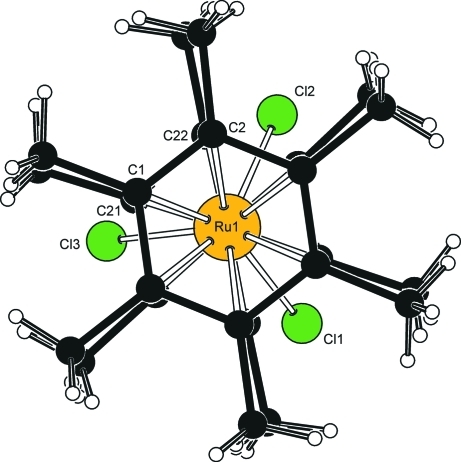
View along the Ru1···Ru2 line of the complex cation in the title compound.

## supplementary crystallographic information

### Comment

A view of the molecular structure of the title compound is given in Fig. 1. The geometry of the complex cation in the structure is very similar to that reported for [(µ–Cl)_3_{Ru(η^6^-C_6_Me_6_)}_2_][PF_6_] (Pandey *et al.*, 1999). The Ru—Cl and Ru—C distances range 2.4354 (6) - 2.4615 (6) Å and 2.170 (2) - 2.192 (3) Å, respectively. The distance between the ruthenium atoms is 3.2723 (3) Å and they are symmetrically bridged by the three chloride ligands with Ru—Cl—Ru angles in the range 83.56 (2)–84.18 (2) °. The π-coordinated arene rings are practically coplanar (dihedral angle of their mean planes is 2.31 (12) °) and assume an almost perfect staggered conformation (Fig. 2).

The [FeCl_4_]^-^ counter ion has a regular tetrahedral geometry with Fe—Cl distances in the range of 2.1890 (8)–2.2018 (10) Å, and interligand angles of 107.10 (3)–110.57 (3) °. These structural data compare well with those observed for simple tetrachloridoferrate(III) salts that have been recently structurally characterized (*e.g.*, quinolinium tetrachloroferrate(III) [Wyrzykowski *et al.*, 2006], and matrinium tetrachloroferrate(III) [Jin *et al.*, 2005]).

In the crystal of the title compound the cations and anions form separate layers that are perpendicular to the crystallographic *a*-axis direction and regularly alternate at distances determined by their van der Waals envelope. No significant hydrogen-bonding interactions (except for some weak C—H···Cl contacts, Table 1) or π···π stacking interactions were detected in the structure, which suggests that the crystal packing is governed predominantly by electrostatic interactions of the ionic constituents.

### Experimental

Burgundy red crystals of the title compound were obtained serendipitously during an attempted crystallization of [(η^6^-C_6_Me_6_)RuCl_2_{Ph_2_PfcCON(CH_2_CH_2_OH)_2_-κ*P*}] (fc = ferrocene-1,1-diyl), which had been  prepared by a conventional bridge-cleavage reaction of [(η^6^-C_6_Me_6_)RuCl_2_]_2_ with Ph_2_PfcCON(CH_2_CH_2_OH)_2_ (Schulz *et al.*, 2009), from chloroform–diethyl ether over an extended period (several weeks). The complex is very likely a decomposition product as the result of photolytic cleavage of the ferrocene moiety in the halogenated solvent (Brand & Snedden, 1957). This has produced FeCl_3_ and chloride ions required for the formation of both the complex cation and complex anion that constitute the title compound.

A few crystals of the product were analysed by electrospray ionization (ESI) mass spectroscopy, which clearly showed signals due to ions [(C_6_Me_6_)_2_Ru_2_Cl_3_]^+^ (*m/z* 633) and [FeCl_4_]^-^ (*m/z* 196) with correct isotopic distribution patterns. High-resolution mass spectra further confirmed the formulation: calculated for [(C_6_Me_6_)_2_^102^Ru_2_^35^Cl_3_]^+^ 632.9970, found 632.9993; calculated for [^56^Fe^35^Cl_4_]^-^ 195.8109, found 195.8108.

### Refinement

All H-atoms were included in their calculated positions and refined as riding atoms: C—H = 0.96 Å, with *U*_iso_(H) = 1.2*U*_eq_(C).

### Crystal data

**Table:**

[Ru_2_Cl_3_(C_12_H_18_)_2_][Cl_4_Fe]	*Z* = 2
*M**_r_* = 830.67	*F*(000) = 826
Triclinic, *P*1	*D*_x_ = 1.839 Mg m^−^^3^
*a* = 8.4490 (2) Å	Mo *K*α radiation, λ = 0.71073 Å
*b* = 12.8352 (2) Å	Cell parameters from 15369 reflections
*c* = 14.6752 (4) Å	θ = 1.0–27.5°
α = 106.5767 (12)°	µ = 2.11 mm^−^^1^
β = 90.4341 (9)°	*T* = 150 K
γ = 99.7915 (12)°	Plate, red
*V* = 1500.43 (6) Å^3^	0.30 × 0.20 × 0.08 mm

### Data collection

**Table:**

Nonius KappaCCD diffractometer	6900 independent reflections
Radiation source: fine-focus sealed tube	6172 reflections with *I* > 2σ(*I*)
horizontally mounted graphite crystal	*R*_int_ = 0.036
Detector resolution: 9.091 pixels mm^-1^	θ_max_ = 27.5°, θ_min_ = 1.5°
ω and π scans to fill the Ewald sphere	*h* = −10→10
Absorption correction: gaussian (Reference? year?)	*k* = −16→16
*T*_min_ = 0.529, *T*_max_ = 0.855	*l* = −19→19
27082 measured reflections	

### Refinement

**Table:**

Refinement on *F*^2^	Primary atom site location: structure-invariant direct methods
Least-squares matrix: full	Secondary atom site location: difference Fourier map
*R*[*F*^2^ > 2σ(*F*^2^)] = 0.026	Hydrogen site location: inferred from neighbouring sites
*wR*(*F*^2^) = 0.061	H-atom parameters constrained
*S* = 1.08	*w* = 1/[σ^2^(*F*_o_^2^) + (0.0211*P*)^2^ + 1.9353*P*] where *P* = (*F*_o_^2^ + 2*F*_c_^2^)/3
6900 reflections	(Δ/σ)_max_ = 0.001
319 parameters	Δρ_max_ = 0.47 e Å^−^^3^
0 restraints	Δρ_min_ = −0.68 e Å^−^^3^

### Special details

**Table:**

Geometry. All e.s.d.'s (except the e.s.d. in the dihedral angle between two l.s. planes) are estimated using the full covariance matrix. The cell e.s.d.'s are taken into account individually in the estimation of e.s.d.'s in distances, angles and torsion angles; correlations between e.s.d.'s in cell parameters are only used when they are defined by crystal symmetry. An approximate (isotropic) treatment of cell e.s.d.'s is used for estimating e.s.d.'s involving l.s. planes.
Refinement. Refinement on F^2^ against all reflections. The weighted R-factor wR and goodness of fit S are based on F^2^, conventional R-factors R are based on F, with F set to zero for negative F^2^. The threshold expression of F^2^ > 2 σ(F^2^) is used only for calculating R-factors(gt) etc. and is not relevant to the choice of reflections for refinement. R-factors based on F^2^ are statistically about twice as large as those based on F, and R- factors based on all data will be even larger.

### Fractional atomic coordinates and isotropic or equivalent isotropic displacement parameters (Å^2^)

**Table:**

	*x*	*y*	*z*	*U*_iso_*/*U*_eq_	
Ru1	0.02214 (2)	0.229351 (15)	0.359389 (13)	0.01330 (5)	
Ru2	−0.10556 (2)	0.289505 (14)	0.172745 (13)	0.01278 (5)	
Cl1	0.01161 (7)	0.13405 (5)	0.18933 (4)	0.02091 (12)	
Cl3	−0.23873 (7)	0.26089 (5)	0.31265 (4)	0.02071 (12)	
Cl2	0.11067 (7)	0.38573 (5)	0.29639 (4)	0.01942 (12)	
C1	−0.0309 (3)	0.2545 (2)	0.50855 (17)	0.0179 (5)	
C2	0.1257 (3)	0.3123 (2)	0.50368 (17)	0.0179 (5)	
C3	0.2415 (3)	0.2553 (2)	0.44795 (17)	0.0175 (5)	
C4	0.1993 (3)	0.1418 (2)	0.39726 (18)	0.0186 (5)	
C5	0.0393 (3)	0.08221 (19)	0.40182 (18)	0.0185 (5)	
C6	−0.0745 (3)	0.1384 (2)	0.45724 (17)	0.0175 (5)	
C7	−0.1569 (3)	0.3129 (2)	0.5627 (2)	0.0252 (6)	
H7A	−0.1180	0.3911	0.5822	0.030*	
H7B	−0.1795	0.2881	0.6179	0.030*	
H7C	−0.2535	0.2965	0.5225	0.030*	
C8	0.1739 (3)	0.4348 (2)	0.5527 (2)	0.0272 (6)	
H8A	0.0814	0.4640	0.5790	0.033*	
H8B	0.2166	0.4717	0.5074	0.033*	
H8C	0.2544	0.4466	0.6029	0.033*	
C9	0.4059 (3)	0.3199 (2)	0.4407 (2)	0.0280 (6)	
H9A	0.4670	0.2723	0.3982	0.034*	
H9B	0.4611	0.3479	0.5026	0.034*	
H9C	0.3940	0.3804	0.4166	0.034*	
C10	0.3173 (3)	0.0828 (2)	0.3344 (2)	0.0282 (6)	
H10A	0.3854	0.1340	0.3087	0.034*	
H10B	0.2593	0.0238	0.2832	0.034*	
H10C	0.3819	0.0531	0.3713	0.034*	
C11	−0.0095 (3)	−0.0368 (2)	0.3438 (2)	0.0270 (6)	
H11A	−0.0604	−0.0791	0.3834	0.032*	
H11B	0.0843	−0.0655	0.3190	0.032*	
H11C	−0.0832	−0.0418	0.2920	0.032*	
C12	−0.2441 (3)	0.0774 (2)	0.4589 (2)	0.0266 (6)	
H12A	−0.2956	0.0551	0.3961	0.032*	
H12B	−0.3039	0.1252	0.5020	0.032*	
H12C	−0.2402	0.0133	0.4795	0.032*	
C21	−0.2956 (3)	0.3670 (2)	0.13151 (18)	0.0178 (5)	
C22	−0.1436 (3)	0.43297 (19)	0.12874 (17)	0.0167 (5)	
C23	−0.0186 (3)	0.38344 (19)	0.07579 (17)	0.0161 (5)	
C24	−0.0481 (3)	0.2687 (2)	0.02469 (17)	0.0179 (5)	
C25	−0.2022 (3)	0.2026 (2)	0.02849 (17)	0.0186 (5)	
C26	−0.3243 (3)	0.2508 (2)	0.08142 (18)	0.0183 (5)	
C27	−0.4253 (3)	0.4163 (2)	0.1900 (2)	0.0247 (6)	
H27A	−0.3818	0.4901	0.2281	0.030*	
H27B	−0.4642	0.3720	0.2308	0.030*	
H27C	−0.5123	0.4180	0.1483	0.030*	
C28	−0.1085 (3)	0.5541 (2)	0.1839 (2)	0.0255 (6)	
H28A	−0.1997	0.5734	0.2192	0.031*	
H28B	−0.0872	0.5976	0.1405	0.031*	
H28C	−0.0162	0.5683	0.2271	0.031*	
C29	0.1449 (3)	0.4543 (2)	0.07921 (19)	0.0234 (5)	
H29A	0.2174	0.4095	0.0445	0.028*	
H29B	0.1857	0.4862	0.1443	0.028*	
H29C	0.1356	0.5120	0.0511	0.028*	
C30	0.0826 (3)	0.2148 (2)	−0.0284 (2)	0.0266 (6)	
H30A	0.1836	0.2649	−0.0119	0.032*	
H30B	0.0580	0.1964	−0.0957	0.032*	
H30C	0.0895	0.1487	−0.0115	0.032*	
C31	−0.2325 (3)	0.0799 (2)	−0.0213 (2)	0.0283 (6)	
H31A	−0.2678	0.0403	0.0235	0.034*	
H31B	−0.1349	0.0585	−0.0472	0.034*	
H31C	−0.3141	0.0629	−0.0718	0.034*	
C32	−0.4824 (3)	0.1789 (2)	0.0883 (2)	0.0275 (6)	
H32A	−0.5410	0.1538	0.0274	0.033*	
H32B	−0.5445	0.2209	0.1347	0.033*	
H32C	−0.4623	0.1163	0.1070	0.033*	
Fe1	0.49209 (4)	0.77416 (3)	0.20266 (3)	0.01901 (8)	
Cl4	0.52218 (8)	0.63784 (5)	0.07833 (5)	0.02709 (14)	
Cl5	0.58902 (10)	0.75020 (7)	0.33345 (6)	0.03875 (18)	
Cl6	0.23261 (7)	0.77493 (5)	0.21100 (5)	0.02774 (14)	
Cl7	0.61347 (8)	0.93196 (5)	0.18546 (5)	0.02927 (15)	

### Atomic displacement parameters (Å^2^)

**Table:**

	*U*^11^	*U*^22^	*U*^33^	*U*^12^	*U*^13^	*U*^23^
Ru1	0.01313 (9)	0.01651 (10)	0.01132 (10)	0.00389 (7)	0.00058 (7)	0.00500 (7)
Ru2	0.01294 (9)	0.01399 (9)	0.01185 (10)	0.00173 (7)	−0.00050 (7)	0.00489 (7)
Cl1	0.0317 (3)	0.0190 (3)	0.0137 (3)	0.0112 (2)	0.0000 (2)	0.0036 (2)
Cl3	0.0141 (3)	0.0338 (3)	0.0193 (3)	0.0068 (2)	0.0028 (2)	0.0143 (3)
Cl2	0.0202 (3)	0.0195 (3)	0.0176 (3)	−0.0020 (2)	−0.0041 (2)	0.0072 (2)
C1	0.0208 (12)	0.0257 (12)	0.0089 (11)	0.0062 (9)	0.0010 (9)	0.0065 (9)
C2	0.0189 (11)	0.0252 (12)	0.0102 (11)	0.0042 (9)	−0.0023 (9)	0.0061 (9)
C3	0.0134 (11)	0.0278 (13)	0.0139 (12)	0.0045 (9)	−0.0019 (9)	0.0096 (10)
C4	0.0175 (11)	0.0239 (12)	0.0186 (13)	0.0086 (9)	−0.0015 (9)	0.0099 (10)
C5	0.0216 (12)	0.0192 (12)	0.0183 (13)	0.0055 (9)	−0.0022 (10)	0.0104 (10)
C6	0.0179 (11)	0.0237 (12)	0.0143 (12)	0.0032 (9)	0.0007 (9)	0.0115 (10)
C7	0.0253 (13)	0.0303 (14)	0.0210 (14)	0.0077 (11)	0.0063 (11)	0.0074 (11)
C8	0.0288 (14)	0.0265 (13)	0.0211 (14)	−0.0001 (11)	−0.0018 (11)	0.0016 (11)
C9	0.0179 (12)	0.0354 (15)	0.0296 (16)	0.0017 (11)	0.0012 (11)	0.0097 (12)
C10	0.0257 (13)	0.0318 (15)	0.0306 (16)	0.0131 (11)	0.0054 (12)	0.0098 (12)
C11	0.0291 (14)	0.0227 (13)	0.0293 (15)	0.0066 (10)	−0.0017 (11)	0.0069 (11)
C12	0.0212 (13)	0.0326 (14)	0.0279 (15)	0.0016 (11)	0.0035 (11)	0.0137 (12)
C21	0.0163 (11)	0.0231 (12)	0.0179 (13)	0.0059 (9)	−0.0031 (9)	0.0111 (10)
C22	0.0211 (11)	0.0167 (11)	0.0161 (12)	0.0047 (9)	−0.0025 (9)	0.0099 (9)
C23	0.0183 (11)	0.0203 (11)	0.0127 (12)	0.0023 (9)	−0.0007 (9)	0.0102 (9)
C24	0.0217 (12)	0.0219 (12)	0.0112 (12)	0.0042 (9)	0.0002 (9)	0.0062 (9)
C25	0.0227 (12)	0.0196 (12)	0.0125 (12)	0.0014 (9)	−0.0037 (9)	0.0044 (9)
C26	0.0159 (11)	0.0231 (12)	0.0163 (12)	0.0002 (9)	−0.0063 (9)	0.0084 (10)
C27	0.0201 (12)	0.0295 (14)	0.0279 (15)	0.0090 (10)	0.0028 (11)	0.0107 (11)
C28	0.0290 (14)	0.0189 (12)	0.0282 (15)	0.0042 (10)	0.0005 (11)	0.0063 (11)
C29	0.0211 (12)	0.0256 (13)	0.0226 (14)	−0.0012 (10)	0.0017 (10)	0.0085 (11)
C30	0.0292 (14)	0.0290 (14)	0.0218 (14)	0.0088 (11)	0.0056 (11)	0.0054 (11)
C31	0.0340 (15)	0.0187 (12)	0.0270 (15)	−0.0003 (11)	−0.0047 (12)	0.0018 (11)
C32	0.0206 (12)	0.0299 (14)	0.0307 (16)	−0.0043 (10)	−0.0035 (11)	0.0121 (12)
Fe1	0.02005 (17)	0.01886 (17)	0.01832 (19)	0.00242 (13)	0.00093 (14)	0.00633 (14)
Cl4	0.0285 (3)	0.0239 (3)	0.0266 (4)	0.0058 (2)	0.0066 (3)	0.0030 (3)
Cl5	0.0465 (4)	0.0444 (4)	0.0288 (4)	0.0040 (3)	−0.0083 (3)	0.0188 (3)
Cl6	0.0224 (3)	0.0257 (3)	0.0344 (4)	0.0054 (2)	0.0061 (3)	0.0068 (3)
Cl7	0.0307 (3)	0.0235 (3)	0.0331 (4)	−0.0034 (3)	−0.0018 (3)	0.0121 (3)

### Geometric parameters (Å, °)

**Table:**

Ru1—C4	2.175 (2)	C10—H10C	0.9600
Ru1—C5	2.177 (2)	C11—H11A	0.9600
Ru1—C6	2.177 (2)	C11—H11B	0.9600
Ru1—C3	2.178 (2)	C11—H11C	0.9600
Ru1—C2	2.180 (2)	C12—H12A	0.9600
Ru1—C1	2.181 (2)	C12—H12B	0.9600
Ru1—Cl3	2.4354 (6)	C12—H12C	0.9600
Ru1—Cl1	2.4393 (6)	C21—C22	1.420 (3)
Ru1—Cl2	2.4498 (6)	C21—C26	1.440 (3)
Ru2—C25	2.170 (2)	C21—C27	1.509 (3)
Ru2—C23	2.171 (2)	C22—C23	1.443 (3)
Ru2—C26	2.175 (2)	C22—C28	1.511 (3)
Ru2—C24	2.181 (2)	C23—C24	1.427 (3)
Ru2—C22	2.188 (2)	C23—C29	1.511 (3)
Ru2—C21	2.192 (2)	C24—C25	1.439 (3)
Ru2—Cl3	2.4374 (6)	C24—C30	1.507 (3)
Ru2—Cl1	2.4425 (6)	C25—C26	1.416 (4)
Ru2—Cl2	2.4616 (6)	C25—C31	1.512 (3)
C1—C2	1.416 (3)	C26—C32	1.511 (3)
C1—C6	1.446 (3)	C27—H27A	0.9600
C1—C7	1.507 (3)	C27—H27B	0.9600
C2—C3	1.441 (3)	C27—H27C	0.9600
C2—C8	1.513 (3)	C28—H28A	0.9600
C3—C4	1.415 (3)	C28—H28B	0.9600
C3—C9	1.513 (3)	C28—H28C	0.9600
C4—C5	1.448 (3)	C29—H29A	0.9600
C4—C10	1.513 (4)	C29—H29B	0.9600
C5—C6	1.421 (3)	C29—H29C	0.9600
C5—C11	1.506 (3)	C30—H30A	0.9600
C6—C12	1.515 (3)	C30—H30B	0.9600
C7—H7A	0.9600	C30—H30C	0.9600
C7—H7B	0.9600	C31—H31A	0.9600
C7—H7C	0.9600	C31—H31B	0.9600
C8—H8A	0.9600	C31—H31C	0.9600
C8—H8B	0.9600	C32—H32A	0.9600
C8—H8C	0.9600	C32—H32B	0.9600
C9—H9A	0.9600	C32—H32C	0.9600
C9—H9B	0.9600	Fe1—Cl4	2.1891 (7)
C9—H9C	0.9600	Fe1—Cl7	2.1925 (7)
C10—H10A	0.9600	Fe1—Cl6	2.1982 (7)
C10—H10B	0.9600	Fe1—Cl5	2.2018 (8)
			
C4—Ru1—C5	38.86 (9)	H7A—C7—H7B	109.5
C4—Ru1—C6	69.45 (9)	C1—C7—H7C	109.5
C5—Ru1—C6	38.09 (9)	H7A—C7—H7C	109.5
C4—Ru1—C3	37.96 (9)	H7B—C7—H7C	109.5
C5—Ru1—C3	69.40 (9)	C2—C8—H8A	109.5
C6—Ru1—C3	81.96 (9)	C2—C8—H8B	109.5
C4—Ru1—C2	69.41 (9)	H8A—C8—H8B	109.5
C5—Ru1—C2	82.33 (9)	C2—C8—H8C	109.5
C6—Ru1—C2	69.31 (9)	H8A—C8—H8C	109.5
C3—Ru1—C2	38.63 (9)	H8B—C8—H8C	109.5
C4—Ru1—C1	82.20 (9)	C3—C9—H9A	109.5
C5—Ru1—C1	69.55 (9)	C3—C9—H9B	109.5
C6—Ru1—C1	38.75 (9)	H9A—C9—H9B	109.5
C3—Ru1—C1	69.14 (9)	C3—C9—H9C	109.5
C2—Ru1—C1	37.89 (9)	H9A—C9—H9C	109.5
C4—Ru1—Cl3	158.47 (7)	H9B—C9—H9C	109.5
C5—Ru1—Cl3	120.10 (7)	C4—C10—H10A	109.5
C6—Ru1—Cl3	94.48 (6)	C4—C10—H10B	109.5
C3—Ru1—Cl3	157.47 (7)	H10A—C10—H10B	109.5
C2—Ru1—Cl3	119.41 (7)	C4—C10—H10C	109.5
C1—Ru1—Cl3	94.18 (6)	H10A—C10—H10C	109.5
C4—Ru1—Cl1	94.63 (7)	H10B—C10—H10C	109.5
C5—Ru1—Cl1	94.10 (7)	C5—C11—H11A	109.5
C6—Ru1—Cl1	119.50 (7)	C5—C11—H11B	109.5
C3—Ru1—Cl1	120.52 (7)	H11A—C11—H11B	109.5
C2—Ru1—Cl1	158.63 (7)	C5—C11—H11C	109.5
C1—Ru1—Cl1	157.61 (7)	H11A—C11—H11C	109.5
Cl3—Ru1—Cl1	80.63 (2)	H11B—C11—H11C	109.5
C4—Ru1—Cl2	119.53 (7)	C6—C12—H12A	109.5
C5—Ru1—Cl2	157.44 (7)	C6—C12—H12B	109.5
C6—Ru1—Cl2	159.55 (7)	H12A—C12—H12B	109.5
C3—Ru1—Cl2	95.09 (6)	C6—C12—H12C	109.5
C2—Ru1—Cl2	95.77 (7)	H12A—C12—H12C	109.5
C1—Ru1—Cl2	121.39 (7)	H12B—C12—H12C	109.5
Cl3—Ru1—Cl2	80.48 (2)	C22—C21—C26	119.8 (2)
Cl1—Ru1—Cl2	79.48 (2)	C22—C21—C27	120.8 (2)
C25—Ru2—C23	69.46 (9)	C26—C21—C27	119.4 (2)
C25—Ru2—C26	38.05 (9)	C22—C21—Ru2	70.92 (13)
C23—Ru2—C26	82.24 (9)	C26—C21—Ru2	70.08 (13)
C25—Ru2—C24	38.62 (9)	C27—C21—Ru2	129.35 (17)
C23—Ru2—C24	38.27 (9)	C21—C22—C23	120.0 (2)
C26—Ru2—C24	69.40 (9)	C21—C22—C28	120.7 (2)
C25—Ru2—C22	81.91 (9)	C23—C22—C28	119.2 (2)
C23—Ru2—C22	38.67 (9)	C21—C22—Ru2	71.24 (13)
C26—Ru2—C22	69.11 (9)	C23—C22—Ru2	70.00 (12)
C24—Ru2—C22	69.42 (9)	C28—C22—Ru2	129.46 (17)
C25—Ru2—C21	69.14 (9)	C24—C23—C22	120.2 (2)
C23—Ru2—C21	69.28 (9)	C24—C23—C29	121.1 (2)
C26—Ru2—C21	38.50 (9)	C22—C23—C29	118.7 (2)
C24—Ru2—C21	82.06 (9)	C24—C23—Ru2	71.26 (13)
C22—Ru2—C21	37.84 (9)	C22—C23—Ru2	71.32 (13)
C25—Ru2—Cl3	122.69 (7)	C29—C23—Ru2	127.77 (17)
C23—Ru2—Cl3	154.96 (7)	C23—C24—C25	119.3 (2)
C26—Ru2—Cl3	95.76 (7)	C23—C24—C30	120.9 (2)
C24—Ru2—Cl3	161.03 (6)	C25—C24—C30	119.7 (2)
C22—Ru2—Cl3	117.41 (7)	C23—C24—Ru2	70.46 (13)
C21—Ru2—Cl3	93.62 (7)	C25—C24—Ru2	70.27 (14)
C25—Ru2—Cl1	93.22 (7)	C30—C24—Ru2	128.70 (18)
C23—Ru2—Cl1	122.67 (7)	C26—C25—C24	120.6 (2)
C26—Ru2—Cl1	117.14 (7)	C26—C25—C31	119.5 (2)
C24—Ru2—Cl1	95.47 (6)	C24—C25—C31	119.9 (2)
C22—Ru2—Cl1	161.18 (7)	C26—C25—Ru2	71.15 (13)
C21—Ru2—Cl1	154.69 (7)	C24—C25—Ru2	71.11 (13)
Cl3—Ru2—Cl1	80.53 (2)	C31—C25—Ru2	128.74 (18)
C25—Ru2—Cl2	154.68 (7)	C25—C26—C21	120.1 (2)
C23—Ru2—Cl2	94.21 (6)	C25—C26—C32	119.7 (2)
C26—Ru2—Cl2	162.53 (7)	C21—C26—C32	120.1 (2)
C24—Ru2—Cl2	117.47 (6)	C25—C26—Ru2	70.80 (13)
C22—Ru2—Cl2	97.49 (6)	C21—C26—Ru2	71.42 (13)
C21—Ru2—Cl2	124.36 (7)	C32—C26—Ru2	128.38 (18)
Cl3—Ru2—Cl2	80.21 (2)	C21—C27—H27A	109.5
Cl1—Ru2—Cl2	79.19 (2)	C21—C27—H27B	109.5
Ru1—Cl1—Ru2	84.181 (19)	H27A—C27—H27B	109.5
Ru1—Cl3—Ru2	84.372 (19)	C21—C27—H27C	109.5
Ru1—Cl2—Ru2	83.559 (18)	H27A—C27—H27C	109.5
C2—C1—C6	119.9 (2)	H27B—C27—H27C	109.5
C2—C1—C7	121.2 (2)	C22—C28—H28A	109.5
C6—C1—C7	118.8 (2)	C22—C28—H28B	109.5
C2—C1—Ru1	71.00 (14)	H28A—C28—H28B	109.5
C6—C1—Ru1	70.48 (13)	C22—C28—H28C	109.5
C7—C1—Ru1	128.64 (17)	H28A—C28—H28C	109.5
C1—C2—C3	119.9 (2)	H28B—C28—H28C	109.5
C1—C2—C8	121.6 (2)	C23—C29—H29A	109.5
C3—C2—C8	118.4 (2)	C23—C29—H29B	109.5
C1—C2—Ru1	71.11 (13)	H29A—C29—H29B	109.5
C3—C2—Ru1	70.61 (13)	C23—C29—H29C	109.5
C8—C2—Ru1	128.84 (17)	H29A—C29—H29C	109.5
C4—C3—C2	120.4 (2)	H29B—C29—H29C	109.5
C4—C3—C9	120.6 (2)	C24—C30—H30A	109.5
C2—C3—C9	118.9 (2)	C24—C30—H30B	109.5
C4—C3—Ru1	70.90 (13)	H30A—C30—H30B	109.5
C2—C3—Ru1	70.76 (13)	C24—C30—H30C	109.5
C9—C3—Ru1	128.73 (18)	H30A—C30—H30C	109.5
C3—C4—C5	120.0 (2)	H30B—C30—H30C	109.5
C3—C4—C10	120.7 (2)	C25—C31—H31A	109.5
C5—C4—C10	119.3 (2)	C25—C31—H31B	109.5
C3—C4—Ru1	71.15 (13)	H31A—C31—H31B	109.5
C5—C4—Ru1	70.65 (13)	C25—C31—H31C	109.5
C10—C4—Ru1	128.39 (18)	H31A—C31—H31C	109.5
C6—C5—C4	119.6 (2)	H31B—C31—H31C	109.5
C6—C5—C11	120.1 (2)	C26—C32—H32A	109.5
C4—C5—C11	120.2 (2)	C26—C32—H32B	109.5
C6—C5—Ru1	70.98 (13)	H32A—C32—H32B	109.5
C4—C5—Ru1	70.49 (13)	C26—C32—H32C	109.5
C11—C5—Ru1	127.97 (18)	H32A—C32—H32C	109.5
C5—C6—C1	120.2 (2)	H32B—C32—H32C	109.5
C5—C6—C12	119.7 (2)	Cl4—Fe1—Cl7	110.26 (3)
C1—C6—C12	120.1 (2)	Cl4—Fe1—Cl6	107.10 (3)
C5—C6—Ru1	70.93 (13)	Cl7—Fe1—Cl6	109.15 (3)
C1—C6—Ru1	70.77 (13)	Cl4—Fe1—Cl5	110.56 (3)
C12—C6—Ru1	128.84 (17)	Cl7—Fe1—Cl5	109.87 (3)
C1—C7—H7A	109.5	Cl6—Fe1—Cl5	109.85 (3)
C1—C7—H7B	109.5		
			
C4—Ru1—Cl1—Ru2	−161.28 (7)	C2—C1—C6—Ru1	−52.9 (2)
C5—Ru1—Cl1—Ru2	159.74 (7)	C7—C1—C6—Ru1	124.1 (2)
C6—Ru1—Cl1—Ru2	129.75 (7)	C4—Ru1—C6—C5	−29.58 (14)
C3—Ru1—Cl1—Ru2	−131.80 (7)	C3—Ru1—C6—C5	−66.69 (14)
C2—Ru1—Cl1—Ru2	−120.87 (18)	C2—Ru1—C6—C5	−104.51 (15)
C1—Ru1—Cl1—Ru2	117.96 (17)	C1—Ru1—C6—C5	−133.2 (2)
Cl3—Ru1—Cl1—Ru2	39.886 (19)	Cl3—Ru1—C6—C5	135.69 (13)
Cl2—Ru1—Cl1—Ru2	−42.073 (19)	Cl1—Ru1—C6—C5	53.93 (15)
C25—Ru2—Cl1—Ru1	−162.48 (7)	Cl2—Ru1—C6—C5	−149.66 (15)
C23—Ru2—Cl1—Ru1	129.94 (7)	C4—Ru1—C6—C1	103.63 (15)
C26—Ru2—Cl1—Ru1	−131.52 (8)	C5—Ru1—C6—C1	133.2 (2)
C24—Ru2—Cl1—Ru1	158.85 (6)	C3—Ru1—C6—C1	66.51 (14)
C22—Ru2—Cl1—Ru1	123.3 (2)	C2—Ru1—C6—C1	28.70 (13)
C21—Ru2—Cl1—Ru1	−118.16 (16)	Cl3—Ru1—C6—C1	−91.10 (13)
Cl3—Ru2—Cl1—Ru1	−39.863 (19)	Cl1—Ru1—C6—C1	−172.86 (11)
Cl2—Ru2—Cl1—Ru1	41.875 (19)	Cl2—Ru1—C6—C1	−16.5 (3)
C4—Ru1—Cl3—Ru2	−118.72 (19)	C4—Ru1—C6—C12	−142.8 (3)
C5—Ru1—Cl3—Ru2	−129.29 (8)	C5—Ru1—C6—C12	−113.2 (3)
C6—Ru1—Cl3—Ru2	−159.16 (7)	C3—Ru1—C6—C12	−179.9 (2)
C3—Ru1—Cl3—Ru2	121.07 (17)	C2—Ru1—C6—C12	142.3 (2)
C2—Ru1—Cl3—Ru2	132.10 (7)	C1—Ru1—C6—C12	113.6 (3)
C1—Ru1—Cl3—Ru2	161.98 (7)	Cl3—Ru1—C6—C12	22.5 (2)
Cl1—Ru1—Cl3—Ru2	−39.971 (19)	Cl1—Ru1—C6—C12	−59.3 (2)
Cl2—Ru1—Cl3—Ru2	40.820 (19)	Cl2—Ru1—C6—C12	97.1 (3)
C25—Ru2—Cl3—Ru1	127.66 (8)	C25—Ru2—C21—C22	−104.19 (16)
C23—Ru2—Cl3—Ru1	−119.47 (15)	C23—Ru2—C21—C22	−29.11 (14)
C26—Ru2—Cl3—Ru1	156.54 (7)	C26—Ru2—C21—C22	−133.2 (2)
C24—Ru2—Cl3—Ru1	119.1 (2)	C24—Ru2—C21—C22	−66.48 (15)
C22—Ru2—Cl3—Ru1	−134.03 (7)	Cl3—Ru2—C21—C22	132.09 (14)
C21—Ru2—Cl3—Ru1	−164.88 (7)	Cl1—Ru2—C21—C22	−152.49 (13)
Cl1—Ru2—Cl3—Ru1	39.923 (19)	Cl2—Ru2—C21—C22	51.49 (16)
Cl2—Ru2—Cl3—Ru1	−40.624 (19)	C25—Ru2—C21—C26	29.01 (14)
C4—Ru1—Cl2—Ru2	131.11 (8)	C23—Ru2—C21—C26	104.09 (16)
C5—Ru1—Cl2—Ru2	116.80 (17)	C24—Ru2—C21—C26	66.72 (15)
C6—Ru1—Cl2—Ru2	−117.51 (18)	C22—Ru2—C21—C26	133.2 (2)
C3—Ru1—Cl2—Ru2	161.87 (7)	Cl3—Ru2—C21—C26	−94.71 (13)
C2—Ru1—Cl2—Ru2	−159.32 (6)	Cl1—Ru2—C21—C26	−19.3 (2)
C1—Ru1—Cl2—Ru2	−129.50 (7)	Cl2—Ru2—C21—C26	−175.31 (11)
Cl3—Ru1—Cl2—Ru2	−40.409 (19)	C25—Ru2—C21—C27	141.2 (2)
Cl1—Ru1—Cl2—Ru2	41.733 (19)	C23—Ru2—C21—C27	−143.7 (2)
C25—Ru2—Cl2—Ru1	−116.04 (15)	C26—Ru2—C21—C27	112.2 (3)
C23—Ru2—Cl2—Ru1	−164.20 (7)	C24—Ru2—C21—C27	178.9 (2)
C26—Ru2—Cl2—Ru1	118.3 (2)	C22—Ru2—C21—C27	−114.6 (3)
C24—Ru2—Cl2—Ru1	−132.30 (7)	Cl3—Ru2—C21—C27	17.5 (2)
C22—Ru2—Cl2—Ru1	157.05 (7)	Cl1—Ru2—C21—C27	92.9 (3)
C21—Ru2—Cl2—Ru1	128.10 (8)	Cl2—Ru2—C21—C27	−63.1 (2)
Cl3—Ru2—Cl2—Ru1	40.409 (19)	C26—C21—C22—C23	0.0 (3)
Cl1—Ru2—Cl2—Ru1	−41.716 (19)	C27—C21—C22—C23	177.3 (2)
C4—Ru1—C1—C2	66.29 (14)	Ru2—C21—C22—C23	52.19 (19)
C5—Ru1—C1—C2	104.32 (15)	C26—C21—C22—C28	−177.5 (2)
C6—Ru1—C1—C2	133.0 (2)	C27—C21—C22—C28	−0.3 (3)
C3—Ru1—C1—C2	29.37 (14)	Ru2—C21—C22—C28	−125.4 (2)
Cl3—Ru1—C1—C2	−135.05 (13)	C26—C21—C22—Ru2	−52.16 (19)
Cl1—Ru1—C1—C2	149.49 (14)	C27—C21—C22—Ru2	125.1 (2)
Cl2—Ru1—C1—C2	−53.67 (15)	C25—Ru2—C22—C21	66.21 (15)
C4—Ru1—C1—C6	−66.71 (14)	C23—Ru2—C22—C21	133.3 (2)
C5—Ru1—C1—C6	−28.68 (13)	C26—Ru2—C22—C21	29.06 (14)
C3—Ru1—C1—C6	−103.63 (15)	C24—Ru2—C22—C21	104.06 (16)
C2—Ru1—C1—C6	−133.0 (2)	Cl3—Ru2—C22—C21	−56.53 (15)
Cl3—Ru1—C1—C6	91.95 (13)	Cl1—Ru2—C22—C21	142.25 (17)
Cl1—Ru1—C1—C6	16.5 (2)	Cl2—Ru2—C22—C21	−139.35 (13)
Cl2—Ru1—C1—C6	173.34 (11)	C25—Ru2—C22—C23	−67.07 (14)
C4—Ru1—C1—C7	−178.5 (2)	C26—Ru2—C22—C23	−104.21 (15)
C5—Ru1—C1—C7	−140.5 (2)	C24—Ru2—C22—C23	−29.21 (13)
C6—Ru1—C1—C7	−111.8 (3)	C21—Ru2—C22—C23	−133.3 (2)
C3—Ru1—C1—C7	144.6 (2)	Cl3—Ru2—C22—C23	170.19 (11)
C2—Ru1—C1—C7	115.2 (3)	Cl1—Ru2—C22—C23	9.0 (3)
Cl3—Ru1—C1—C7	−19.9 (2)	Cl2—Ru2—C22—C23	87.38 (13)
Cl1—Ru1—C1—C7	−95.3 (3)	C25—Ru2—C22—C28	−179.0 (2)
Cl2—Ru1—C1—C7	61.5 (2)	C23—Ru2—C22—C28	−111.9 (3)
C6—C1—C2—C3	−0.3 (3)	C26—Ru2—C22—C28	143.9 (2)
C7—C1—C2—C3	−177.3 (2)	C24—Ru2—C22—C28	−141.1 (2)
Ru1—C1—C2—C3	−53.0 (2)	C21—Ru2—C22—C28	114.8 (3)
C6—C1—C2—C8	177.3 (2)	Cl3—Ru2—C22—C28	58.3 (2)
C7—C1—C2—C8	0.3 (4)	Cl1—Ru2—C22—C28	−103.0 (3)
Ru1—C1—C2—C8	124.6 (2)	Cl2—Ru2—C22—C28	−24.5 (2)
C6—C1—C2—Ru1	52.7 (2)	C21—C22—C23—C24	1.2 (3)
C7—C1—C2—Ru1	−124.3 (2)	C28—C22—C23—C24	178.8 (2)
C4—Ru1—C2—C1	−104.29 (15)	Ru2—C22—C23—C24	53.92 (19)
C5—Ru1—C2—C1	−66.36 (15)	C21—C22—C23—C29	−176.3 (2)
C6—Ru1—C2—C1	−29.30 (14)	C28—C22—C23—C29	1.3 (3)
C3—Ru1—C2—C1	−132.8 (2)	Ru2—C22—C23—C29	−123.5 (2)
Cl3—Ru1—C2—C1	53.98 (15)	C21—C22—C23—Ru2	−52.75 (19)
Cl1—Ru1—C2—C1	−147.94 (15)	C28—C22—C23—Ru2	124.8 (2)
Cl2—Ru1—C2—C1	136.28 (13)	C25—Ru2—C23—C24	−29.30 (14)
C4—Ru1—C2—C3	28.48 (14)	C26—Ru2—C23—C24	−66.40 (14)
C5—Ru1—C2—C3	66.42 (14)	C22—Ru2—C23—C24	−132.5 (2)
C6—Ru1—C2—C3	103.47 (15)	C21—Ru2—C23—C24	−103.94 (15)
C1—Ru1—C2—C3	132.8 (2)	Cl3—Ru2—C23—C24	−153.40 (13)
Cl3—Ru1—C2—C3	−173.25 (11)	Cl1—Ru2—C23—C24	50.96 (15)
Cl1—Ru1—C2—C3	−15.2 (3)	Cl2—Ru2—C23—C24	130.80 (13)
Cl2—Ru1—C2—C3	−90.95 (13)	C25—Ru2—C23—C22	103.17 (15)
C4—Ru1—C2—C8	139.9 (2)	C26—Ru2—C23—C22	66.07 (14)
C5—Ru1—C2—C8	177.8 (2)	C24—Ru2—C23—C22	132.5 (2)
C6—Ru1—C2—C8	−145.1 (2)	C21—Ru2—C23—C22	28.52 (13)
C3—Ru1—C2—C8	111.4 (3)	Cl3—Ru2—C23—C22	−20.9 (2)
C1—Ru1—C2—C8	−115.8 (3)	Cl1—Ru2—C23—C22	−176.57 (11)
Cl3—Ru1—C2—C8	−61.9 (2)	Cl2—Ru2—C23—C22	−96.73 (13)
Cl1—Ru1—C2—C8	96.2 (3)	C25—Ru2—C23—C29	−144.6 (2)
Cl2—Ru1—C2—C8	20.4 (2)	C26—Ru2—C23—C29	178.4 (2)
C1—C2—C3—C4	0.6 (3)	C24—Ru2—C23—C29	−115.2 (3)
C8—C2—C3—C4	−177.1 (2)	C22—Ru2—C23—C29	112.3 (3)
Ru1—C2—C3—C4	−52.7 (2)	C21—Ru2—C23—C29	140.8 (2)
C1—C2—C3—C9	177.6 (2)	Cl3—Ru2—C23—C29	91.4 (2)
C8—C2—C3—C9	−0.1 (3)	Cl1—Ru2—C23—C29	−64.3 (2)
Ru1—C2—C3—C9	124.3 (2)	Cl2—Ru2—C23—C29	15.6 (2)
C1—C2—C3—Ru1	53.2 (2)	C22—C23—C24—C25	−1.5 (3)
C8—C2—C3—Ru1	−124.4 (2)	C29—C23—C24—C25	175.8 (2)
C5—Ru1—C3—C4	29.47 (14)	Ru2—C23—C24—C25	52.4 (2)
C6—Ru1—C3—C4	66.72 (15)	C22—C23—C24—C30	−178.1 (2)
C2—Ru1—C3—C4	133.5 (2)	C29—C23—C24—C30	−0.7 (4)
C1—Ru1—C3—C4	104.62 (16)	Ru2—C23—C24—C30	−124.1 (2)
Cl3—Ru1—C3—C4	148.97 (15)	C22—C23—C24—Ru2	−53.94 (19)
Cl1—Ru1—C3—C4	−52.89 (15)	C29—C23—C24—Ru2	123.4 (2)
Cl2—Ru1—C3—C4	−133.66 (13)	C25—Ru2—C24—C23	132.8 (2)
C4—Ru1—C3—C2	−133.5 (2)	C26—Ru2—C24—C23	104.08 (15)
C5—Ru1—C3—C2	−104.00 (15)	C22—Ru2—C24—C23	29.50 (13)
C6—Ru1—C3—C2	−66.75 (14)	C21—Ru2—C24—C23	66.43 (14)
C1—Ru1—C3—C2	−28.85 (14)	Cl3—Ru2—C24—C23	144.33 (17)
Cl3—Ru1—C3—C2	15.5 (3)	Cl1—Ru2—C24—C23	−138.95 (12)
Cl1—Ru1—C3—C2	173.64 (11)	Cl2—Ru2—C24—C23	−58.30 (14)
Cl2—Ru1—C3—C2	92.87 (13)	C23—Ru2—C24—C25	−132.8 (2)
C4—Ru1—C3—C9	114.4 (3)	C26—Ru2—C24—C25	−28.67 (14)
C5—Ru1—C3—C9	143.9 (3)	C22—Ru2—C24—C25	−103.26 (15)
C6—Ru1—C3—C9	−178.9 (2)	C21—Ru2—C24—C25	−66.33 (14)
C2—Ru1—C3—C9	−112.1 (3)	Cl3—Ru2—C24—C25	11.6 (3)
C1—Ru1—C3—C9	−141.0 (3)	Cl1—Ru2—C24—C25	88.30 (13)
Cl3—Ru1—C3—C9	−96.6 (3)	Cl2—Ru2—C24—C25	168.94 (11)
Cl1—Ru1—C3—C9	61.5 (2)	C25—Ru2—C24—C30	−112.8 (3)
Cl2—Ru1—C3—C9	−19.2 (2)	C23—Ru2—C24—C30	114.5 (3)
C2—C3—C4—C5	−0.5 (3)	C26—Ru2—C24—C30	−141.4 (2)
C9—C3—C4—C5	−177.4 (2)	C22—Ru2—C24—C30	144.0 (2)
Ru1—C3—C4—C5	−53.1 (2)	C21—Ru2—C24—C30	−179.1 (2)
C2—C3—C4—C10	176.7 (2)	Cl3—Ru2—C24—C30	−101.2 (3)
C9—C3—C4—C10	−0.3 (4)	Cl1—Ru2—C24—C30	−24.5 (2)
Ru1—C3—C4—C10	124.1 (2)	Cl2—Ru2—C24—C30	56.2 (2)
C2—C3—C4—Ru1	52.6 (2)	C23—C24—C25—C26	0.7 (3)
C9—C3—C4—Ru1	−124.4 (2)	C30—C24—C25—C26	177.3 (2)
C5—Ru1—C4—C3	−132.8 (2)	Ru2—C24—C25—C26	53.2 (2)
C6—Ru1—C4—C3	−103.75 (16)	C23—C24—C25—C31	−177.0 (2)
C2—Ru1—C4—C3	−28.95 (14)	C30—C24—C25—C31	−0.4 (4)
C1—Ru1—C4—C3	−65.87 (15)	Ru2—C24—C25—C31	−124.5 (2)
Cl3—Ru1—C4—C3	−147.43 (16)	C23—C24—C25—Ru2	−52.5 (2)
Cl1—Ru1—C4—C3	136.43 (13)	C30—C24—C25—Ru2	124.1 (2)
Cl2—Ru1—C4—C3	55.91 (15)	C23—Ru2—C25—C26	−104.17 (15)
C6—Ru1—C4—C5	29.03 (14)	C24—Ru2—C25—C26	−133.2 (2)
C3—Ru1—C4—C5	132.8 (2)	C22—Ru2—C25—C26	−66.25 (14)
C2—Ru1—C4—C5	103.83 (16)	C21—Ru2—C25—C26	−29.33 (14)
C1—Ru1—C4—C5	66.91 (15)	Cl3—Ru2—C25—C26	51.22 (15)
Cl3—Ru1—C4—C5	−14.7 (3)	Cl1—Ru2—C25—C26	132.03 (13)
Cl1—Ru1—C4—C5	−90.79 (14)	Cl2—Ru2—C25—C26	−156.68 (13)
Cl2—Ru1—C4—C5	−171.31 (12)	C23—Ru2—C25—C24	29.06 (13)
C5—Ru1—C4—C10	112.5 (3)	C26—Ru2—C25—C24	133.2 (2)
C6—Ru1—C4—C10	141.6 (2)	C22—Ru2—C25—C24	66.98 (14)
C3—Ru1—C4—C10	−114.7 (3)	C21—Ru2—C25—C24	103.90 (15)
C2—Ru1—C4—C10	−143.6 (2)	Cl3—Ru2—C25—C24	−175.55 (11)
C1—Ru1—C4—C10	179.5 (2)	Cl1—Ru2—C25—C24	−94.75 (13)
Cl3—Ru1—C4—C10	97.9 (3)	Cl2—Ru2—C25—C24	−23.4 (2)
Cl1—Ru1—C4—C10	21.8 (2)	C23—Ru2—C25—C31	142.7 (2)
Cl2—Ru1—C4—C10	−58.8 (2)	C26—Ru2—C25—C31	−113.1 (3)
C3—C4—C5—C6	0.1 (3)	C24—Ru2—C25—C31	113.7 (3)
C10—C4—C5—C6	−177.1 (2)	C22—Ru2—C25—C31	−179.4 (2)
Ru1—C4—C5—C6	−53.21 (19)	C21—Ru2—C25—C31	−142.4 (2)
C3—C4—C5—C11	176.6 (2)	Cl3—Ru2—C25—C31	−61.9 (2)
C10—C4—C5—C11	−0.6 (3)	Cl1—Ru2—C25—C31	18.9 (2)
Ru1—C4—C5—C11	123.3 (2)	Cl2—Ru2—C25—C31	90.2 (3)
C3—C4—C5—Ru1	53.3 (2)	C24—C25—C26—C21	0.5 (3)
C10—C4—C5—Ru1	−123.9 (2)	C31—C25—C26—C21	178.2 (2)
C4—Ru1—C5—C6	132.6 (2)	Ru2—C25—C26—C21	53.7 (2)
C3—Ru1—C5—C6	103.72 (15)	C24—C25—C26—C32	−177.1 (2)
C2—Ru1—C5—C6	66.04 (14)	C31—C25—C26—C32	0.6 (3)
C1—Ru1—C5—C6	29.14 (14)	Ru2—C25—C26—C32	−123.9 (2)
Cl3—Ru1—C5—C6	−53.60 (15)	C24—C25—C26—Ru2	−53.2 (2)
Cl1—Ru1—C5—C6	−135.14 (13)	C31—C25—C26—Ru2	124.5 (2)
Cl2—Ru1—C5—C6	152.61 (15)	C22—C21—C26—C25	−0.8 (3)
C6—Ru1—C5—C4	−132.6 (2)	C27—C21—C26—C25	−178.1 (2)
C3—Ru1—C5—C4	−28.83 (14)	Ru2—C21—C26—C25	−53.4 (2)
C2—Ru1—C5—C4	−66.51 (15)	C22—C21—C26—C32	176.8 (2)
C1—Ru1—C5—C4	−103.42 (16)	C27—C21—C26—C32	−0.5 (3)
Cl3—Ru1—C5—C4	173.84 (12)	Ru2—C21—C26—C32	124.2 (2)
Cl1—Ru1—C5—C4	92.30 (14)	C22—C21—C26—Ru2	52.55 (19)
Cl2—Ru1—C5—C4	20.0 (3)	C27—C21—C26—Ru2	−124.7 (2)
C4—Ru1—C5—C11	−113.6 (3)	C23—Ru2—C26—C25	66.39 (15)
C6—Ru1—C5—C11	113.8 (3)	C24—Ru2—C26—C25	29.07 (14)
C3—Ru1—C5—C11	−142.5 (2)	C22—Ru2—C26—C25	104.08 (15)
C2—Ru1—C5—C11	179.8 (2)	C21—Ru2—C26—C25	132.7 (2)
C1—Ru1—C5—C11	142.9 (2)	Cl3—Ru2—C26—C25	−138.75 (13)
Cl3—Ru1—C5—C11	60.2 (2)	Cl1—Ru2—C26—C25	−56.45 (15)
Cl1—Ru1—C5—C11	−21.3 (2)	Cl2—Ru2—C26—C25	145.66 (19)
Cl2—Ru1—C5—C11	−93.6 (3)	C25—Ru2—C26—C21	−132.7 (2)
C4—C5—C6—C1	0.2 (3)	C23—Ru2—C26—C21	−66.28 (15)
C11—C5—C6—C1	−176.3 (2)	C24—Ru2—C26—C21	−103.61 (15)
Ru1—C5—C6—C1	−52.80 (19)	C22—Ru2—C26—C21	−28.60 (14)
C4—C5—C6—C12	177.5 (2)	Cl3—Ru2—C26—C21	88.57 (13)
C11—C5—C6—C12	1.0 (3)	Cl1—Ru2—C26—C21	170.87 (12)
Ru1—C5—C6—C12	124.5 (2)	Cl2—Ru2—C26—C21	13.0 (3)
C4—C5—C6—Ru1	52.98 (19)	C25—Ru2—C26—C32	113.2 (3)
C11—C5—C6—Ru1	−123.5 (2)	C23—Ru2—C26—C32	179.6 (2)
C2—C1—C6—C5	−0.1 (3)	C24—Ru2—C26—C32	142.2 (3)
C7—C1—C6—C5	177.0 (2)	C22—Ru2—C26—C32	−142.8 (3)
Ru1—C1—C6—C5	52.9 (2)	C21—Ru2—C26—C32	−114.2 (3)
C2—C1—C6—C12	−177.4 (2)	Cl3—Ru2—C26—C32	−25.6 (2)
C7—C1—C6—C12	−0.3 (3)	Cl1—Ru2—C26—C32	56.7 (2)
Ru1—C1—C6—C12	−124.4 (2)	Cl2—Ru2—C26—C32	−101.2 (3)

### Hydrogen-bond geometry (Å, °)

**Table:**

*D*—H···*A*	*D*—H	H···*A*	*D*···*A*	*D*—H···*A*
C9—H9A···Cl3^i^	0.96	2.80	3.629 (3)	145
C11—H11B···Cl6^ii^	0.96	2.71	3.588 (3)	153

Symmetry codes: (i) *x*+1, *y*, *z*; (ii) *x*, *y*−1, *z*.
